# Tumor Cell Dormancy and Reactivation in Bone: Skeletal Biology and Therapeutic Opportunities

**DOI:** 10.1002/jbm4.10125

**Published:** 2019-01-07

**Authors:** Niall M Byrne, Matthew A Summers, Michelle M McDonald

**Affiliations:** ^1^ Bone Biology Division The Garvan Institute of Medical Research Darlinghurst NSW Sydney Australia; ^2^ St Vincent's Clinical School Faculty of Medicine, UNSW Sydney Darlinghurst NSW Australia

**Keywords:** BONE MICROENVIRONMENT, BONE METASTASIS, DORMANCY, REACTIVATION, OSTEOCLAST, ANTIRESORPTIVES

## Abstract

In the advanced stages of many cancers, tumor cells disseminate from the primary site and colonize distant locations such as the skeleton. These disseminated tumor cells colonizing bone can evade treatments and survive for prolonged periods in a dormant state before becoming reactivated to form overt metastases. The precise interactions between tumor cells and the bone microenvironment that promote survival, dormancy, and reactivation are currently unknown; as a result, bone metastases remain incurable. In this review we discuss the unique cellular and microenvironmental features of endosteal bone that tumor cells engage with to persist and survive, and ultimately reactivate and proliferate. Specifically, we provide a detailed summary of current perspectives on the processes of tumor cell colonization of the skeleton, and the endosteal bone cells as critical controllers of the dormant cancer cell phenotype, as well as relevant microenvironmental effects such as hypoxia. Evidence for the role of the osteoclast in controlling dormant cancer cell reactivation in bone is highlighted, preceding a discussion of therapeutics targeting the bone microenvironment, including anti‐RANK ligand and bisphosphonate therapies and their potential utility in preventing tumor cell reactivation in addition to protecting bone from tumor‐induced destruction. © 2018 The Authors. *JBMR Plus* published by Wiley Periodicals, Inc. on behalf of American Society for Bone and Mineral Research.

## Introduction

For a number of cancers the skeleton is a frequent site for metastasis, this is particularly true of breast (BCa) and prostate cancer (PCa), and the hematological malignancy, multiple myeloma (MM), which often develops in bone.^1^, ^2^ Although treatment options and survival outcomes have significantly improved for most cancers including localized disease,^3^ skeletal metastasis is often associated with significant morbidity^4^, ^5^, ^6^ and mortality^7^, ^8^, ^9^—with current therapeutics offering only modest survival benefits.^10^ Furthermore, the incidence of bone metastatic PCa has increased over the last decade and is expected to rise again within the next 10 years, highlighting the critical need for more effective therapies for skeletal metastatic disease.^11^, ^12^

The development of skeletal metastasis is a complex, multistep process, and significant advances have been made in the understanding of this cascade including translational and mathematical models of skeletal disease.^13^, ^14^, ^15^ This metastatic process usually begins with *intravasation* of tumor cells from the primary site into the lymphatic or vasculature system, likely through the process of epithelial‐to‐mesenchymal transition (EMT), before *extravasation* of these disseminated tumor cells (DTCs) into compatible secondary sites that support their growth. Stephen Paget first postulated a site‐specific compatibility in 1889 when he described the unique association between cancers of the breast and secondary growths in bone.^16^ Paget's “seed and soil” hypothesis argued that the environment (“soil”) at secondary sites plays a critical role in the success or failure of cancer cells (“seeds”) to survive and thrive.

In bone metastatic disease, only a limited number of DTCs survive following colonization of bone, and are retained in a dormant state through engagement in specialized “niches” in the bone microenvironment. These rare, dormant tumor cells are thought to initiate bone metastasis upon reactivation and contribute to disease recurrence.^13^, ^17^, ^18^, ^19^ Defining the niches in bone, the control of tumor cell dormancy and reactivation are arguably the most fundamental questions in understanding the initiation of bone metastatic disease and in designing therapies that selectively target tumor cells in the skeleton.

In this review we first discuss the early events in tumor cell colonization of bone, and the critical role of the bone microenvironment in regulating tumor cell dormancy. Though “dormancy” can be used to describe different physiological observations, herein it will be used to define quiescent or slow‐cycling single tumor cells. We consider the events that lead to reactivation of these dormant tumor cells within the skeleton and discuss the translational models and preclinical studies that have helped define these mechanisms. Whereas cell‐intrinsic mechanisms and immune regulation may play a role in controlling tumor cell dormancy, for the purpose of this review we will focus on the tumor cell‐extrinsic environmental signals that may govern this process, as the former has been discussed elsewhere.^20^, ^21^ We conclude with future directions to further profile dormant tumor cells and the metastatic niches in bone, and discuss the clinical implications and therapeutic opportunities of bone‐targeted agents for treating skeletal metastasis.

## Tumor Cell Dormancy in Bone and Niche Engagement

Although bone metastasis represents a hallmark of advanced disease, the establishment of tumors at secondary sites is an inefficient process: DTCs can be detected in the bones of patients with PCa and BCa; however, not all patients develop metastatic disease.^22^, ^23^, ^24^, ^25^ Furthermore, it is not uncommon for women with BCa to go through a period of extended latency before the onset of metastatic growth.^26^ Taken together, this suggests that DTCs can colonize skeletal sites early in the development of the disease,^27^ but that there are a number of challenges that must be overcome for these cells to survive and prosper.^28^

### Tumor cell colonization of the skeleton

The first of the challenges in the metastatic cascade is the homing and extravasation of DTCs in the circulation to the bone marrow. A number of factors are thought to have a role in attracting tumor cells to the bone microenvironment, including the CXCL12/CXCR4 signaling axis.^29^ The chemoattractant CXCL12 (C‐X‐C motif chemokine ligand 12) is expressed on osteoblasts, and endothelial and bone marrow stromal cells, including mesenchymal stem/stromal cells (MSCs). It is also involved in the recruitment of hematopoietic stem cells (HSCs) to the bone marrow through its receptor CXCR4 (C‐X‐C motif chemokine receptor 4).^30^, ^31^ Expression of CXCR4 has been shown to promote invasion and metastasis in melanoma, BCa, and PCa.^32^, ^33^ This may be mediated through its effector protein phosphatidylinositol 4‐kinase IIIα (PI4KIIIα), which localizes to lipid rafts with CXCR4, contributing to tumor cell homing to metastatic sites and subsequent invasion.^34^ Furthermore, inhibition of the CXCL12–CXCR4 axis has been shown to compromise the establishment of PCa tumors in bone, while having no effect on pre‐established tumors in the skeleton.^35^ The local expression of other molecules, including integrins such as αvβ3, can facilitate adhesion and anchorage of DTCs to the extracellular matrix (ECM) of the bone microenvironment^36^ and promotes BCa metastasis to bone.^37^ Additionally, the expression of αvβ3 in PCa cells is enhanced by CXCL12/CXCR4 binding.^38^ The osteoblast‐derived WISP‐1 has recently been implicated in the adhesion of PCa cells to osteoblasts via the VCAM‐1/integrin α4β1 system.^39^

In addition, other secreted factors such as matrix metalloproteinases (MMPs) can prime the microenvironment prior to the arrival of DTCs, remodeling bone to create a “premetastatic niche” amenable to metastatic dissemination.^40^, ^41^ In fact, secretion of lysyl oxidase (LOX) from distal BCa tumors has been shown to induce focal osteolytic lesions in bone, deregulating normal bone homeostasis to create a premetastatic niche.^42^, ^43^ The concept of the premetastatic niche in tumor progression has been extensively reviewed elsewhere.^44^

### The endosteal bone microenvironment and tumor cell dormancy

Following DTC homing to bone, only a limited number of DTCs survive. This hypothesis is supported by intravital imaging of MM cells arriving in bone; of these only a few will progress to form overt tumors.^13^ Colonizing DTCs can localize to specific niches in the endosteal bone microenvironment supporting their survival, immune evasion, and the acquisition of a dormant phenotype associated with increased chemoresistance.^13^, ^19^ Although the composition of these metastatic niches remains incompletely characterized, there is evidence that PCa cells compete for the HSC niche, with MSCs and cells of the osteoblast lineage promoting prolonged quiescence.^45^ Furthermore, BCa and PCa cells have been observed favoring homing to osteoblast‐rich regions in bone.^18^, ^46^

Whereas the molecular mechanisms that underpin the control of tumor cell dormancy remain largely uncharacterized, specific ligand/receptor interactions with osteoblasts are a suggested mechanism. Notably, the ligand growth arrest‐specific‐6 (GAS6) binding the protein tyrosine kinase receptor MER is implicated in lymphoblastic leukemia cell dormancy in bone,^47^ and a GAS6/AXL interaction with osteoblasts has been shown to hold PCa cells in a dormant state.^48^ PCa cells have been shown to express a repertoire of these GAS6 receptors including AXL, MER, and TYRO3; similar to the regulation of HSC quiescence, a balance in the expression of these receptors controls dormancy (AXL) or proliferation (TYRO3).^49^ Conditioned media from differentiated osteoblasts, but not undifferentiated osteoblasts, have been shown to induce PCa cell quiescence in vitro. This was reported to be through the osteoblast‐secreted factors growth differentiation factor 10 (GDF10) and transforming growth factor‐beta 2 (TGFβ2), which induced PCa tumor cell dormancy in bone through activation of the signaling pathway TGFβRIII–p38MAPK–pS249/pT252–RB.^47^, ^50^ Furthermore, lower levels of TGFβRIII were found to correlate with metastatic progression and a worse clinical outcome in PCa patients.^50^

Exosomes derived from bone marrow MSCs (BM‐MSCs) have also been proposed in maintaining tumor cell dormancy: culture of human BCa cells with exosomes isolated from BM‐MSCs suppressed BCa cell proliferation and decreased their sensitivity to chemotherapy.^51^ Interestingly, BCa cells have been shown to prime MSCs to release exosomes containing microRNAs to promote quiescence.^52^ Furthermore, BCa cells may acquire dormancy inducing microRNAs through cannibalizing MSCs.^53^ Taken together, this would suggest that cells of the osteoblast lineage are a critical component of the metastatic cascade and dormancy in bone; however, it remains unclear in vivo whether these are MSCs, bone‐lining cells, or terminally differentiated osteoblasts. Equally, other myeloid or endothelial populations may play a role in the initial stages of tumor engraftment and dormancy.

### Perivascular niche and oxygen tensions in the bone microenvironment

The microvasculature and endothelial cells of the bone marrow have been implicated in tumor cell dormancy in models of BCa metastasis and HSC quiescence.^54^, ^55^, ^56^ Dormant BCa cells were found closely aligned with stable bone microvasculature associated with the expression of thrombospondin‐1, and sprouting neovascularization was found to accelerate tumor growth through TGF‐β1 and periostin release from endothelial tip cells.^55^ 3D imaging and computational modeling of the murine BM determined that quiescent HSCs aligned with small arterioles encircled with NG2‐positive pericytes in the endosteal BM; deletion of NG2‐positive cells resulted in the induction of HSC cycling.^57^ In murine models of BCa metastasis, concomitant inhibition of vascular endothelial growth factor (VEGF) signaling and production reduced skeletal tumor burden and the development of osteolytic lesions.^58^ However, the involvement of the vasculature at the early stages of the metastatic cascade is unknown, as treatment was initiated after the development of bone metastasis.

Although bone is a highly vascularized tissue, oxygen tensions vary across the marrow cavity, and endosteal and periosteal surfaces. Recent intravital imaging studies in animals have observed tissue oxygenation to be quite low across the BM. This was potentiated in deeper sinusoidal regions with local oxygenation as low as approximately 1.3% (9.9 mm Hg), whereas in contrast oxygenation increased closer to the endosteal regions of bone approximately 1.8% (13.5 mm Hg)^59^; physiological oxygen levels in normal tissue are in the range of 4% to 7.5%.^60^ The fluctuating oxygen tensions across bone may have implications for the recruitment and long‐term dormancy of DTCs.^61^ The adaptive response to changes in tissue oxygenation is mediated through hypoxia‐inducible factors (HIFs): a heterodimer consisting of an oxygen‐dependent α‐subunit and stable β‐subunit. HIF‐1 is one of the most studied, and quiescent HSCs have been found to maintain stabilized HIF‐1α.^62^ Stabilization of HIF‐1α in MM cells by the tripartite motif TRIM44 was found to contribute to cellular quiescence and survival in hypoxia. TRIM44 was reported to be upregulated in quiescent MM cells in the BM in comparison with reactivated cells.^63^ Furthermore, CXCL12 expression is elevated in hypoxic tissue through HIF‐1, leading to the recruitment of CXCR4‐positive progenitor cells^64^; therefore, extravasation of CXCR4‐positve DTCs into the bone marrow may be supported by a hypoxic milieu.

In DTCs isolated from bone marrow specimens from BCa patients, Grp78‐positive stress granules were observed consistent with the likelihood these cells were exposed to acute hypoxic cell stress.^65^ The interleukin‐6 (IL‐6) cytokine leukemia inhibitory factor (LIF) has also been implicated in maintaining BCa tumor cell dormancy in bone through its receptor LIFR via LIFR:STAT3:SOCS3 signaling.^66^, ^67^ Conversely, in hypoxic regions within bone, STAT3 signaling is downregulated (associated with increased tumor invasion in bone), and may provide a mechanism for the reactivation of dormant cells.^67^ Therefore, although low oxygen tension (hypoxia) is certainly a characteristic of the bone microenvironment, its role in DTC colonization, tumor dormancy, and reactivation is less clear. In addition, chemotherapeutics and radiation have also been shown to manipulate local tissue oxygenation in the bone microenvironment.^59^ This could alter the supportive niches in which dormant tumor cells reside, subsequently inducing their reactivation and growth in the skeleton.

## Dormant Tumor Cell Reactivation in Bone

How dormant tumor cells become reactivated, or how the bone microenvironment relinquishes control of these cells after an extended period remains poorly understood. Intravital imaging of the bone microenvironment in murine models of MM has shown that dormant tumor cells can exist even in the presence of reactivated proliferating cells,^13^ suggesting relevant clonal heterogeneity exists within DTC populations, and that dormant cells may represent a discrete clonal subtype. However, to date, evidence for cell intrinsic dormancy pathways is lacking, whereas more substantial evidence suggests these pathways are triggered by microenvironmental cues.^68^

### Environmental control of reactivation

Manipulation of the bone microenvironment through increasing bone turnover has frequently been shown to increase metastatic burden and frequency of overt tumors in the skeleton. In experimental models, increasing bone turnover, whether induced through castration,^17^ ovariectomy,^69^, ^70^ stimulation with parathyroid hormone (PTH),^71^ or calcium restriction,^72^ resulted in increased tumor development in bone. In addition, the effects reported could be ablated using inhibitors of resorption such as OPG or bisphosphonates.^17^, ^69^, ^73^, ^74^

In models of PCa and BCa the incidence of overt bone metastasis is increased in young mice (where bone turnover is elevated) in comparison with mature mice, while having no effect on nonskeletal tumor development; however, DTCs could still be detected in the bones of mature animals despite the reduced tumor growth.^17^, ^69^, ^75^ Androgen deprivation therapy (ADT), the mainstay of treatment for men with PCa, is commonly associated with bone loss^76^ and when bone turnover was elevated in mature mice through ADT (castration), the frequency of overt PCa bone metastasis increased.^17^ A similar effect was reported in models of BCa following ovariectomy‐induced bone turnover, suggesting that changes to the bone microenvironment are sufficient to reactivate dormant tumor cells in the skeleton.^69^ ADT has also been associated with remodeling the vasculature and inducing hypoxia in primary PCa xenografts.^77^, ^78^ Although its effect on the microarchitecture of the bone vasculature is less well‐characterized, angiogenesis and bone resorption are coupled through MMP9, which may have implications for the perivascular niche and dormant cell reactivation.^79^

As mentioned previously, osteoblastic conditions may induce dormancy, implicating increased osteoclast activity in the reactivation of dormant cells in those scenarios with high bone turnover. Indeed, intravital imaging of colonizing DTCs in models of MM have shown that soluble receptor activator of nuclear factor kappa‐Β ligand‐ (sRANKL‐) driven increases in osteoclastic bone resorption released dormant tumor cells from the endosteal bone niche, while having no effect on DTCs in soft tissue sites including the spleen.^13^ This mechanism is not without precedent, and is similar to mechanisms of dormant HSC mobilization in the bone microenvironment.^80^ This has major implications for treatments that are known to modify bone turnover.

### Consequences of dormant tumor cell reactivation

Although dormant DTC reactivation is the proposed mechanism for tumor development in the skeleton, it is not unreasonable to assume that metastatic sites seed other metastatic sites.^81^, ^82^, ^83^ In fact, these events need not be mutually exclusive, with DTCs from one metastatic site entering dormancy at another, before a reactivation event occurs. Nevertheless, following reactivation DTCs can become proliferative, leading to the formation of overt metastases. Significant progress has been made in characterizing the later stages of the metastatic cascade, including the environment control and modifying events associated with overt tumor growth in the skeleton, in what is often regarded as the “vicious cycle.” Herein, tumor cells can stimulate bone formation or resorption, and in turn the microenvironment releases factors that further stimulate tumor growth. For example, tumor‐derived parathyroid hormone‐related protein (PTHrP) stimulates osteoclastogenesis and subsequent bone resorption through the cytokine RANKL that binds to its receptor (RANK) on the surface of osteoclasts and its precursors. This osteoclastic bone resorption in turn leads to the release of TGFβ from the bone extracellular matrix, which has direct effects stimulating tumor growth.^84^, ^85^, ^86^, ^87^ At this stage of metastasis, the disease is largely incurable; it is likely that early intervention is required for treatments to succeed, prior to the reactivation of dormant tumor cells.

## Therapeutic Opportunities to Prevent or Exploit Reactivation

Current treatment regimens for bone metastatic disease are designed to induce tumor regression, inhibit further tumor growth and progression, or target tumor‐associated skeletal events that define the latter stages of the disease (including pathological fracture and bone pain). Given the importance of osteoclasts and osteoblasts in controlling the fate of tumor cells in the early stages of the disease, there are implications for agents that directly or indirectly affect the bone microenvironment. However, this also highlights unique therapeutic opportunities that may be gained by exploiting existing therapeutics. Dormant tumor cells could be reactivated in bone for subsequent eradication by chemotherapy, or their reactivation prevented through suppressing bone resorption by osteoclasts, which will be the focus of this review.

### Targeting bone turnover to reactivate dormant tumor cells

Clinically used therapies that are known to increase bone turnover may reactivate dormant tumor cells in bone. For example, androgen‐ and estrogen‐targeted therapies, as discussed above, stimulate bone cell activity and therefore have the potential to release dormant cells from their niche. In addition, the alkylating drug melphalan, which is used to treat patients with MM, has been shown to induce osteoclast formation and modify the bone microenvironment^88^; thus it has the potential to reactivate dormant tumor cells. Although to date no clinical studies have demonstrated such negative implications of these agents, informed scheduling of these approaches have the potential to allow for total eradication of chemoresistant, dormant tumor cells in bone. Further opportunities may also be gained from targeting mechanisms of niche engagement or dormancy‐related molecules including AXL or TGFβ2 signaling (as discussed above) to reactivate dormant tumor cells for eradication using currently available chemotherapeutics.^19^

### Targeting the bone microenvironment to suppress reactivation

Inhibitors of bone resorption such as bisphosphonates or anti‐RANKL denosumab (DMab) are standards of care to prevent tumor‐induced bone destruction and fractures, and exert their effects through targeting osteoclasts. Given the evidence thus far highlighted, it can be predicted that these drugs are also likely to limit the reactivation of dormant cells by inhibiting bone resorption. In doing so, these agents could suppress tumor outgrowth in bone in the first instance and during disease recurrence, thereby prolonging life.

In the context of PCa, the evidence for tumor growth effects independent of skeletal‐related events (SRE) is limited. Several clinical studies using the bisphosphonate zoledronic acid (ZOL) have shown reduced time to first SRE,^89^, ^90^ and reduced overall SRE occurrence^91^, ^92^ concurrent with ADT treatments. However, some have found no effects,^93^ and none has as yet directly assessed bisphosphonates effects on limiting bone metastasis following ADT. In contrast, patients with MM treated with ZOL were found to have improved overall survival independent of effects on SRE occurrence,^94^, ^95^ suggesting direct effects on dormant tumor cell reactivation and growth in this context.

In BCa patients, the evidence for tumor effects is far greater. For example, results from the Austrian Breast and Colorectal Cancer Study Group Trial 12 found that ZOL improved patient survival when combined with endocrine therapy in premenopausal women with early stage BCa.^96^, ^97^, ^98^ In the AZURE trial (Chemotherapy and/or Hormone Therapy With or Without Zoledronate in Treating Women With Stage II or Stage III Breast Cancer), ZOL had no benefit to disease‐free survival time, though it did improve outcomes and reduce the development of bone metastasis.^99^ This was consistent with the findings of the Early Breast Cancer Trialists’ Collaborative Group, showing the use of adjuvant bisphosphonates in early BCa reduced the rate of metastatic bone recurrence and improved BCa survival.^100^ Additionally, recent use of the bisphosphonate alendronate in postmenopausal women with early BCa was also associated with a lower likelihood of bone metastasis.^101^ Collectively, this evidence has led to the development of new clinical guidelines from international groups, including the American Society of Clinical Oncology and the European Society for Medical Oncology, recommending adjuvant bisphosphonate therapy in stages II and III BCa patients who are postmenopausal to prevent cancer recurrence.^102^, ^103^

Similar effects in the prevention of SREs have been reported with the use of the anti‐RANKL agent DMab, which interrupts the RANK–RANKL signaling involved in osteoclastogenesis. In castration‐resistant prostate cancer (CRPC) patients with metastatic disease at onset of treatment, DMab significantly delayed the time to first SRE, and has been found to be superior to ZOL in reducing the risk of SREs.^104^, ^105^ In patients with metastatic BCa, DMab treatment reduced the occurrence of SREs through increasing BMD.^97^, ^106^ Furthermore, in patients with bone metastases from solid tumors (other than BCa and PCa), DMab was superior to ZOL in delaying first on study SREs.^107^ In the same study, in MM patients DMab achieved efficacy with ZOL in preventing on study SREs. In addition to its improvements in bone mass and prevention of SREs, data are now emerging to determine whether DMab also has the capacity to improve metastasis‐free survival as an indication of its capacity to inhibit dormant cell reactivation.

DMab was found to significantly increase bone metastasis‐free survival in CRPC patients with no evidence of bone metastasis,^105^ and has also been shown to have the greatest efficacy in men with CRPC at high risk for progression.^108^ However, the effect of DMab in men with castration‐sensitive PCa and bone metastasis is currently unknown, as is the effect of DMab when initiated concomitant to ADT in these individuals. Similarly, in the context of BCa, a placebo‐controlled phase III clinical trial revealed a significant increase in DFS in patients with HR positive BCa treated with aromatase inhibitors.^109^ Of particular interest, early data from a recent phase III clinical trial with the primary outcome to assess metastasis‐free survival in patients with early BCa showed that the time to on‐study fracture and time to bone metastases as first recurrence were reduced.^110^ Conversely in this study, unlike bisphosphonate, DMab did not reduce the number of BCa recurrences in bone nor increase disease‐free survival. Further analysis of these and other ongoing studies will determine whether DMab has the capacity to delay the recurrence of bone metastatic disease and hence be approved for preventive treatment in high‐risk patients without bone marrow metastases.

### Clinical considerations for treatment

Taken together these clinical studies suggest that informed scheduling of inhibitors of bone resorption may prevent the reactivation of dormant tumor cells in the skeleton before the development of overt metastatic disease. However, consideration must be given to the emerging evidence for a rapid rebound loss in bone mass following DMab withdrawal in patients with osteoporosis when treating bone cancer patients.^111^ In a phase II trial, within 6 to 12 months of treatment cessation, serum CTX levels rose to twice the placebo levels and lumbar spine BMD returning to baseline levels.^112^ Similar findings were demonstrated in a phase III prevention study^113^ and an extension of the FREEDOM (A Study to Evaluate Denosumab in the Treatment of Postmenopausal Osteoporosis) trial.^114^ Although these studies have not clearly demonstrated a subsequent increased risk of fracture, experts in the field are suggesting the cancellation of DMab holidays, or advising in the case treatment cessation is required, that bisphosphonate treatment is applied to prevent rebound bone loss.^115^, ^116^ Given the rapid bone loss, a pathological increase in osteoclast activity is the likely cause and has been suggested in small cohort studies through serum analyses,^117^ although the mechanism for this remains to be determined. Any rapid and pathological increase in osteoclast activity could be detrimental in the bone metastatic patient, increasing their risk of fracture and morbidity. Moreover, dormant tumor cells reactivated by aberrant osteoclast activity during this rebound window could increase the risk of disease recurrence and thereby impact survival. Therefore, care must be taken in situations where DMab treatment is withdrawn in patients with bone metastatic disease.

## Conclusions and Future Directions

It is evident that upon arrival in bone, tumor cells engage with a multitude of local cells that support their engraftment, survival, and growth, but also regulate their capacity to maintain dormancy. The reactivation of dormant tumor cells within the skeleton is a complex process that is regulated, at least in part, by tumor cell‐extrinsic mechanisms including osteoclastic bone resorption. Recent technological advances in intravital imaging and single cell sequencing are likely to improve our understanding of the early events in bone metastatic disease, including the mechanisms for reactivation of dormant tumor cells. These findings may highlight novel targets for therapeutic interventions, which will ultimately eradicate skeletal tumors. Meanwhile, clinical data are emerging to suggest antiresorptive agents have the capacity to not only reduce bone loss and fractures, but also prolong life by suppressing tumor growth in bone, potentially via the inhibition of osteoclast‐driven dormant tumor cell reactivation. The evidence supporting this in the context of bisphosphonates has led to international advocacy for the use of adjuvant therapy in early BCa patients to prevent bone metastatic disease. The use of DMab in this setting has not yet been advocated, given the data surrounding its impact on disease‐free survival are limited and at times conflicting. In fact, the first large cohort study (Study of Denosumab as Adjuvant Treatment for Women With High Risk Early Breast Cancer Receiving Neoadjuvant or Adjuvant Therapy [D‐CARE]) aimed at assessing metastatic growth, a disease‐free survival benefit was not reported. This suggests that the differences in the mechanisms of action between ZOL and DMab could differentially impact dormant tumor cell reactivation. Although the mechanism behind this differential response remains to be determined, a direct comparison of these two classes of antiresorptive agents with the occurrence or recurrence of bone metastatic growth as a primary outcome may unveil this mechanism. Even so, this body of work highlights the potential to utilize our capacity to regulate bone cell behavior to prevent the development of metastatic bone cancers.

## Disclosures

Authors have no conflicts of interest to declare.
